# 

**Published:** 1909-08

**Authors:** 


### Contributors to Vol. LXV.

AUGUST, 1909—JULY, 1910.

---

Adler, Lewis H., Jr. . . . . . . . . . . . . . . . . . . . . . . . . . . . Philadelphia
Beahan, A. L. . . . . . . . . . . . . . . . . . . . . . . . . . . . . . . Canandaigua
Billings, William H. . . . . . . . . . . . . . . . . . . . . . . . . . . . Buffalo
Bowerman, Edwin A. . . . . . . . . . . . . . . . . . . . . . . . . . . . Buffalo
Brown, William M. . . . . . . . . . . . . . . . . . . . . . . . . . . . Rochester
Cannaday, J. E. . . . . . . . . . . . . . . . . . . . . . . . Charleston, W. Va.
Chase, Walter B. . . . . . . . . . . . . . . . . . . . . . . . . . . . . . Brooklyn
Conway, M. P. . . . . . . . . . . . . . . . . . . . . . . . . . . . . . . Auburn
Creveling, J. P. . . . . . . . . . . . . . . . . . . . . . . . . . . . . . Auburn
Danforth, W. C. . . . . . . . . . . . . . . . . . . . . . . . . . . Evanston, Ill.
Dowd, J. Henry . . . . . . . . . . . . . . . . . . . . . . . . . . . . . . Buffalo
Frost, Henry F. . . . . . . . . . . . . . . . . . . . . . . . . . . . . . . Buffalo
Goodyear, A. C. . . . . . . . . . . . . . . . . . . . . . . . . . . . . . . Buffalo
Gram, Franklin C. . . . . . . . . . . . . . . . . . . . . . . . . . . . . . Buffalo
Hartel, Fritz . . . . . . . . . . . . . . . . . . . . . . . . . . . . . . . . Berlin
Hayd, Herman E. . . . . . . . . . . . . . . . . . . . . . . . . . . . . . . Buffalo
Hays, Harold . . . . . . . . . . . . . . . . . . . . . . . . . . . . . . . New York
Hirst, B. C. . . . . . . . . . . . . . . . . . . . . . . . . . . . . . Philadelphia
Hopkins, Henry R. . . . . . . . . . . . . . . . . . . . . . . . . . . . . . Buffalo
Jacobson, Nathan . . . . . . . . . . . . . . . . . . . . . . . . . . . . Syracuse
King, James E. . . . . . . . . . . . . . . . . . . . . . . . . . . . . . . Buffalo
Knopf, S. A. . . . . . . . . . . . . . . . . . . . . . . . . . . . . . . New York
Lucid, M. M. . . . . . . . . . . . . . . . . . . . . . . . . . . . . . . Cortland
McGuire, Francis W. . . . . . . . . . . . . . . . . . . . . . . . . . . . Buffalo
Mann, Matthew D. . . . . . . . . . . . . . . . . . . . . . . . . . . . . . Buffalo
Meisenbach, R. O. . . . . . . . . . . . . . . . . . . . . . . . . . . . . Buffalo
Motter, Murray Galt . . . . . . . . . . . . . . . . . . . . . . . . . Washington

Owen, Robert L. .......................... Washington
Putnam, James Wright .......................... Buffalo
Rafter, John A. .......................... Buffalo
Reed, Charles A. L. .......................... Cincinnati
Spofford, H. M. .......................... Batavia
Stephenson, F. H. .......................... Syracuse
Tinker, William B. .......................... Ithaca
Underhill, Eugene .......................... Philadelphia
Van Peyma, P. W. .......................... Buffalo
Wallace, W. L. .......................... Syracuse
Young, J. C. .......................... Cuba

American Gynecological Society
American Proctologic Society
Buffalo Academy of Medicine
Chicago Medical Society
Medical Society County of Erie
National Confederation State Medical Examining and Licensing Boards

### BOOKS RECEIVED.

Clinical Treatises on the Pathology and Therapy of Disorders of Metabolism and Nutrition. Part VIII. Gout, by Professor Dr. H. Strauss, Professor of the Third Clinic, Royal Charity Hospital, Berlin. Authorised American Edition, translated under the direction of Nellis Barnes Foster, M.D., Associate Physician, New York Hospital, etc., New York: E. B. Treat & Company. 1909. (Price, $1.00.)

Clinical Treatises on the Symptomatology and Diagnosis of Disorders of Respiration and Circulation. By Professor Edmund von Neusser, M.D., Professor of the Second Medical Clinic, Vienna; Associate Editor of Nothnagel's Practice of Medicine. Authorised translation by Andrew MacFarlane, M.D., Professor of Medical Jurisprudence and Physical Diagnosis at the Albany Medical College, etc. Part III, Angina Pectoris, New York: E. B. Treat & Company. 190[illegible]. (Price, $1.00.)

Dorland's American Illustrated Medical Dictionary. A new and complete dictionary of terms used in Medicine, Surgery, Dentistry, Pharmacy, Chemistry, Nursing, and kindred branches; with new and elaborate tables and many handsome illustrations. Fifth revised edition. By W. A. Newman Dorland, M.D., large octavo of 876 pages, with 2,000 new terms. Philadelphia and London. W. B. Saunders Company, 1909. (Flexible leather, $4.50 net; indexed, $5.00 net.)

Bier's Hyperemic Treatment in Surgery, Medicine and all the Specialties: A Manual of Its Practical Application. By Willy Meyer, M.D., Professor of Surgery at the New York Post-Graduate Medical School and Hospital: and Professor Dr. Victor Schmieden, Assistant to Professor Bier at Berlin University, Germany. Second revised edition. Octavo of 280 pages, illustrated. Philadelphia and London: W. B. Saunders Company, 1909. (Cloth, $3.00 net.)

Exercise in Education and Medicine. By R. Tait McKenzie, A.B., M.D., Professor of Physical Education, and Director of the Department, University of Pennsylvania. Octavo of 406 pages, with 346 illustrations. Philadelphia and London: W. B. Saunders Company, 1909. (Cloth, $3.50 net; half-morocco, $5.00 net.)

The Priniciples of Pharmacy. By Henry V. Arny, Ph.G., Ph.D., Professor of Pharmacy at the Cleveland School of Pharmacy, Pharmacy Department of Western Reserve University. Octavo of 1,175 pages, with 246 illustrations, mostly original. Philadelphia and London: W. B. Saunders Company, 1909. Cloth, $5.00 net; half-morocco, $6.50 net.)

Tuberculosis, a Preventable and Curable Disease. Modern methods for the solution of the tuberculosis problem, by S. Adolphus Knopf, M.D., Professor of Phthisiotherapy at the New York Post-graduate Medical School and Hospital. Associate Director of the Clinic for Pulmonary Diseases of the Health Department, etc. New York: Moffat, Yard & Company. 1909.

Human Physiology, an Elementary Textbook of Anatomy, Physiology and Hygiene. By John W. Ritchie, Professor of Biology, at the College of William and Mary, Virginia. Illustrated by Mary H. Wellman. Yonkers-on-Hudson, N. Y.: World Book Company. 1909.

Rational Immunisation in the Treatment of Pulmonary Tuberculosis and other diseases, comprising a paper read before the Royal Society of Medicine, March, 1909. By E. C. Hort, B.A., B.Sc., M.R.C.S. New York: William Wood and Company. 1909.

The Ophthalmic Year Book, Volume VI., containing a Digest of the Literature of Ophthalmology with index of publications for the year 1908. By Edward Jackson, A.M., M.D., Professor of Ophthalmology at the University of Colorado; George de Schweinitz, A.M., M.D., Professor of Ophthalmology at the University of Pennsylvania; and Theodore B. Schmeideman, A.M., M.D., Professor of Ophthalmology at the Philadelphia Polyclinic. Illustrated. The Herrick Book and Stationery Company, Denver, Colo. 1909.

A Theory Regarding the Origin of Cancer. By C. E. Green. Second edition. Edinburgh and London: William Green & Sons. 1909.

The Practical Medicine Series. Comprising ten volumes on the year's progress in medicine and surgery, under the general charge of Gustavus P. Head, M.D., professor Laryngology and Rhinology at the Chicago Postgraduate Medical School, Vol. V. Obstetrics. Edited by Joseph B. DeLee, A.M., M.D., Professor of Obstetrics at the Northwestern University Medical School, with the Collaboration of Herbert M. Stowe, M.D. Series of 1909. Chicago: The Year Book Publishers. 1909.

